# HIV testing experiences in Nairobi slums: the good, the bad and the ugly

**DOI:** 10.1186/s12889-019-7975-7

**Published:** 2019-11-29

**Authors:** Eliud Wekesa

**Affiliations:** Department of Sociology, Anthropology and Community Development, South Eastern Kenya University, P.O Box 170-90200, Kitui, Kenya

**Keywords:** HIV testing, VCT, PITC, PLWHA, Slums, Kenya, sub-Saharan Africa

## Abstract

**Background:**

HIV testing is an integral component of HIV prevention, treatment and care and, therefore, is crucial in achieving UNAIDS 90–90-90 targets. HIV testing in Kenya follows both the voluntary counselling and testing (VCT) and provider initiated testing and counselling (PITC) models. However, little is known about the individual experiences of undergoing an HIV test in the two testing models. This study provides experiential evidence of undergoing an HIV test in a resource poor urban slum setting.

**Methods:**

The study explored testing experiences and challenges faced in respect to ensuring the 3 Cs (consent, counselling and confidentiality), using in-depth interviews (*N* = 41) with HIV-infected men and women in two slum settlements of Nairobi City. The in-depth interview respondents were aged above 18 years with 56% being females. All interviews were audio-recorded, transcribed and then translated into English. The transcribed data were analysed using thematic analysis method.

**Results:**

The respondent HIV-testing experiences were varied and greatly shaped by circumstances and motivation for HIV testing. The findings show both positive and negative experiences, with sporadic adherence to the 3Cs principle in both HIV testing models. Although some respondents were satisfied with the HIV testing process, a number of them raised a number of concerns, with instances of coercion and testing without consent being reported.

**Conclusion:**

The 3Cs (consent, counselling and confidentiality) principle must underlie HIV testing and counselling practices in order to achieve positive testing outcomes. The study concludes that adherence to the 3Cs during HIV testing contributes to both the individual and public health good – irrespective of whether testing is initiated by the individual or by the health provider.

## Background

HIV/AIDS disproportionately affects sub-Saharan Africa, which accounts for nearly 70% of the 36.9 million global burden [1]. In 2017, there were an estimated 25.7 million people living with HIV, 1.8 new infections and about 1.0 million HIV/AIDS related death in sub-Saharan Africa (SSA) [1]. In Kenya in excess of 1.5 million adults are living with HIV, accounting for a prevalence rate of 4.9% [2], with urban slums residents having higher rates [3]. In response to the HIV pandemic in this part of the world, prevention efforts have increasingly placed an emphasis on early HIV testing and identification of HIV positive individuals [4]. HIV testing has received considerable promotion, not least because it is a gateway to treatment and provision of prevention messages. It is, thus, considered an integral part of a comprehensive package of universal prevention, treatment and care [5]. That is why it is an important pillar in the current 90–90-90 goal by the UNAIDs, which aims to accelerate progress towards ending HIV/AIDS by 2030 [6]. Consequently, the prevailing HIV prevention discourse is, understandably, framed around the Seek, Test, Treat and Retain mantra [7, 8].

HIV testing practises, principles and guidelines in Kenya (as elsewhere in sub-Saharan Africa) have been changing in response to low testing rates and the increasing availability of antiretroviral treatment (ART). In the early stages of the HIV epidemic, testing was done under the framework of voluntary counselling and testing (VCT). Self-initiated voluntary testing is the hallmark of the VCT model [9]. In this approach the testing is done at the initiative of the individual to be tested, usually by voluntarily visiting a VCT centre [10]. It has been suggested that VCT follows an “opt in” approach following intensive counselling; pre-test counselling is a prerequisite, hence C preceding T [11]. Counsellors are supposed to ensure informed consent prior to the test so that people undergoing the test are ready to deal with the HIV test outcome [12]. Voluntary counselling and testing was introduced in sub-Saharan Africa in the 1990s, with international guidelines developed by UNAIDS [11]. However, despite promotional efforts, uptake of VCT is relatively low and only 1/5 of Kenyan adults get tested in VCT centres [13].

To increase testing rates and access to antiretroviral treatment (ART), policy makers, researchers and international guidelines recommended an alternative HIV testing approach: routine provider-initiated HIV testing and counselling (PITC). In the PITC approach, the onus of initiating the test rests with the health provider, rather than the individual client. In contrast to VCT, PITC follows an “opt-out” approach [11], where the provider initiates and performs the HIV test unless the patient declines [14]. The emphasis in PITC is placed on testing before counselling thus, T preceding C [11]. The PITC approach has been credited with recent increases in HIV testing rates and linkages to treatment and care in low and middle income countries [15]. In Kenya, PITC has been rolled out since 2005 and is implemented predominantly by nurses using rapid tests, albeit not without challenges [16]. The co-existence of the VCT and PITC HIV testing models in Kenya has seen adult HIV testing rates more than double from 34% in 2007 to 71% in 2012 [17].

The changing of HIV testing protocols and consequent increases in HIV testing rates notwithstanding, there remains a lingering debate between “opt-in” (VCT) and “opt-out” (PITC) testing proponents. On one hand, the VCT approach proponents and fierce critics of the PITC model are represented by Csete and Elliot [18]. Their stand takes the human right stance, which underscores the cardinal principle of minimizing harm and maximizing benefits. They argue that the principle of 3 Cs – consent (informed and verbal), counselling (pre and post), and confidentiality (of seeking test and test result) – of the policy statement on HIV testing [9] – can only be guaranteed under the VCT model. Their central argument against the PITC is that it violates the individual rights of those being tested and does not minimize harm and maximize benefits [18].

On the other hand, the PITC advocates, who place precedence of the public health right over individual human rights are represented by De Cock and colleagues [19]. Their central premise is that the traditional VCT model is an exemplification of the HIV/AIDS “exceptionalism”, which in addition to fuelling stigma, is an impediment to effective prevention and treatment responses. They contend that the VCT model is too slow an approach to be used for a public health emergency such as HIV/AIDS. They, therefore, argue that routine provider-initiated testing will normalise HIV testing procedure and make it similar with other diseases, thereby reducing stigma, increasing testing uptake and detection of HIV, and linkages to treatment. They argue that consent is implicit in their (clients) seeking care and does not need to be stated clearly prior to testing.

In this article, I contribute to this policy debate, not by denouncing one or the other testing model, but by presenting lived-experience data on the actual practice of HIV testing in the two testing models. I examine the experiential process of testing in both the VCT and PITC regimes and assess the extent to which their respective guidelines are adhered to in actual practice in slums of Nairobi. There is an information gap on the experience of the actual practice of HIV testing in sub-Saharan Africa and challenges faced in resource-poor settings such as slums. Most studies on HIV testing in SSA have mainly been quantitative and thus have focused on the HIV testing uptake/ testing rates and their determinants [20–23]. A few qualitative studies have, on their part concentrated on its benefits, costs and barriers [24, 25]. There is, therefore, a dearth of information on lived-experience of the process, circumstances and people’s views of undergoing an HIV test. Information on the experience of testing is necessary to understand how HIV testing guidelines are implemented in practice, which is essential for policy and programme improvement of HIV testing services [26]. Moreover, the contexts and circumstances under which a person gets tested have a bearing on subsequent sexual and reproductive health (SRH) behaviour. Evidence shows that HIV testing and counselling (HCT) is associated with adoption of preventive sexual behaviour [27, 28].

## Methods

### Study setting

The data for this paper is derived from a qualitative study conducted as part of a larger mixed method study of people living with HIV/AIDS (PLWHA) in the two slums (Korogocho and Viwandani) of Nairobi in 2010. The two slums are located in the Nairobi Urban Demographic Surveillance System run by the African Population and Health Research Centre. The living conditions in the two slum settlements are poor, with houses typically consisting of single rooms, constructed from mud, iron sheets, cardboard boxes and polythene. The slums settlements are often illegal and thus informal and, therefore, government authorities are reluctant to provide social amenities and services such as schools, roads, and healthcare facilities. The two slums are characterized by inadequate access to formal health, education and other social services. The combined effects of poverty, inadequate access to health and social services, and poor environmental conditions place residents of the two slums at heightened risk of HIV infection, risky sexual behaviour, early sexual initiation and early childbearing and morbidity and mortality [3, 29].

### Study design and selection of participants

The study design involved a quantitative survey phase (*N* = 503), which was followed by a qualitative phase with a subsample of survey respondents. Forty-one (41) semi-structured qualitative interviews were conducted with individual HIV-positive men and women. The quantitative survey respondents provided a sampling frame for the subsequent in-depth interviews. In-depth interviewees were purposely selected on the basis of their socio-demographic characteristics, socio-behavioural and sexual and reproductive health outcomes as captured in the quantitative survey. In-depth interviewees were thus systematically selected to ensure wide socio-demographic (gender, age, marital status and ethnicity) and socio-behavioural outcomes (ART use, sexual activity, fertility preferences and contraceptive use) representation from the quantitative sample. Consequently, 45 individuals were identified to undergo in-depth interview; 41 interviews were conducted (three had moved and we were unable to trace them, one refused to be re-interviewed). The detailed research design is described elsewhere [30].

### Data collection procedure

Four (2 female and 2 male) trained local interviewers conducted face-to-face qualitative interviews in Kiswahili in February 2010. Written informed consent was obtained from all participants prior to the interviews. Interviews were conducted in private locations chosen by the interviewees, took approximately 1 h to complete and covered a wide range of individual HIV experiences, including HIV testing services, disclosure, and treatment.

### Data management and analysis

All interviews were audio-recorded, transcribed verbatim in kiswahili and then translated into English. The transcripts were read several times to identify themes and categories. A coding frame was developed and the transcripts coded using inductive coding in NVivo 9.0 and analyzed thematically. Codes were grouped into categories and themes were generated on the basis of interpreting the underlying meaning of categories. A coding scheme comprising the main theme, sub-themes and exemplary quotes was developed, and this was expanded as new themes emerged following the systematic reading and coding of transcripts.

## Results

The background characteristics of the qualitative study participants are summarised in Table 1. A total of 41 women and men living with HIV/AIDS participated in the qualitative study; the majority of which (56%) were female. More than half (51%) were young adults aged below 40 and less than half (44%) were on ART. Other summary statistics of the socio-demographic characteristics of the qualitative sample are described in Table 1.

**Table 1:** Percentage distribution of socio-demographic characteristics in-depth interviewees (n-41) living with HIV/AIDS in Nairobi slums

Characteristic	Respondents	
	Number(N)	Percentage (%)
Slum residence		
Korogocho	23	56
Viwandani	18	44
Sex of respondent		
Female	23	56
Male	18	44
Age in years		
18–29	8	20
30–39	13	31
40 and more	20	49
Marital status		
Married/cohabiting	12	29
Divorced/Separated	9	22
Widowed	14	34
Never Married	6	15
Ethnicity		
Kikuyu	15	37
Luo	11	27
Luyia	7	17
Kamba	5	12
Other	3	7
Living Children		
0	2	5
1–2	16	39
3 to 4	14	34
5 and more	9	22
Treatment status		
On ART (Ref)	18	44
Not on ART	23	56

Detailed analyses of findings are presented along two broad subheadings; namely: 1.Circumstances and motivations of HIV testing; and 2. the actual HIV-testing experiences (the good, the bad and the ugly).

### HIV testing: circumstances and motivations

During our interviews, PLWHA reported a variety of circumstances and motivations for undergoing an HIV test. The two most common reasons given by PLWHA for undergoing the HIV test is that they requested an HIV test following frequent illness and recurrence of symptoms (25/41), and that an HIV test was offered during the course of a routine antenatal testing or health care visit (10/41).

*INTE: How did you come to know your status?*

*VC03: I came to learn about it in December 2006. I suspected myself after a series of illness episodes and then decided to go for the HIV test. I used to suffer from fatigue, often feeling like I was running out of energy. I was also suffering from frequent bouts of malaria, headaches, fever, joint pains and feeling nauseated. There were also sores in my mouth. That is why I went for medical advice (cohabiting woman aged 36).*

A number (8/23) of women respondents came to learn of their HIV status when they went for antenatal care. According to testing protocols, pregnant women in Kenya are offered an HIV test as part of their antenatal care. In this case the HIV test is done at the same time as other routine antenatal tests. This is intended to prevent mother to child transmission of HIV because in the absence of any intervention an HIV positive woman can pass the virus on to her baby during pregnancy, birth or breast milk.

*INTE: Let’s now talk about how you learnt of your HIV Status.*

*VB06: It was in 2007 when I was pregnant with my last child. It [pregnancy] was two months old. I had gone to confirm if indeed I was pregnant at matter hospital. They tested my urine and confirmed that I was pregnant. Then they asked me if I could go to the VCT and I agreed. The results turned out to be HIV positive. (Cohabiting woman, aged 35)*

Testing during antenatal care was one of the routes by which spouses came to know their HIV status. Women who tested positive were sometimes asked to bring their spouses to the clinic for HIV testing. PMTCT service guidelines in Kenya call for couple testing and partner involvement in the services [31].

Other common motivations included perceiving themselves at risk for HIV infection especially upon having a family member test positive for HIV. Some respondents explained that having an intimate partner diagnosed with HIV affected their HIV risk perceptions and decisions to get tested. Such respondents began to question their HIV status when their former or current sexual partners died of HIV-related conditions or suspicious circumstances.

*INTE: When did you get to know that you were HIV positive?*

*VC11: After my first wife died of AIDS in 2005, I thought on my own volition to go and get tested just in case I had the [HIV] virus … because you know nowadays you could be sick and fail to know what you are suffering from. Then later on I sweet-talked my other wife to also go and get tested and she agreed and we got tested. (married man aged 46)*

The transcripts also revealed that they also wanted to know their status if their spouses became sickly or started experiencing recurrent sickness that are associated with HIV/AIDS.

### HIV-testing experience: the good

Both the provider and client-initiated HIV testing in Kenya share three core principles: Consent, confidentiality and counselling (3CS). The guidelines in Kenya prescribe that HIV testing must be accompanied by pre-test and post-test counselling (Ministry of Health 2008b). Several participants explained that they were tested in compliance with the HIV testing guidelines and protocols. Narratives of those who went on their own volition to the VCT centres almost exclusively paint a picture of receiving counselling prior to testing. Some respondents expressed satisfaction with their VCT experience.

*(VB10): I found another doctor who counselled me very well and I agreed to go for the VCT. When the doctor counselled me, I got encouraged and well prepared for the outcome of the VCT. I was tested and found to be positive. The result did not surprise me at all because she [doctor] had made me see how life would continue even if I was found to be positive. So when they confirmed it [HIV], I just said it was fine. As long as there are drugs, I would just continue with (taking) drugs, but I did not worry much. The fear I had initially had gone after being counselled well. (Married man aged 43)*

The contents of comprehensive HIV counselling revolve around general information about the HIV condition, the meaning of a test result, preparing the client to receive the test, and how to conduct themselves thereafter, whether they test positive or negative. Some of the information given during counselling included how to live with HIV and other sexual and reproductive health matters

*INTE: Were you counselled before taking the test?*

*VC03: The nurse counselled me and told me about the availability of drugs that people with HIV were using. She told me that if I took the drugs well and avoided frequent sexual contact with men, I would live for long, and sure enough I have lived all this while thanks to that. She also advised me to always use condoms. (Cohabiting woman aged 36)*

Testing, even when self-initiated, is not an autonomous individual voluntary decision; it is often done in response to some triggers such as illness or prompts from healthcare workers and significant others. That is, arguably, one of the main reasons why individual-based VCT model has not raised the level of testing in SSA as nearly 80% of HIV infected adults in SSA are unaware of their status despite VCT promotion [4].

### HIV testing: the bad

Fears abound that the routine provider-initiated HIV testing may not guarantee all the 3 three tenets: consent, confidentiality and counselling. Some respondents reported lack of adherence to the 3 principles prior to testing. Some respondents who had gone to seek healthcare for a different illness reported that informed consent was not sought and given before they were tested for HIV.

*VA13: I was tested for HIV in 2007 when I went to hospital for treatment for a different disease.*

*INTE: What exactly did he [doctor] tell you before testing?*

*VA13: No, he didn’t tell me anything. I was tested everything, urine and blood, but not told anything. Just a letter to Dr. [name withheld] for further action (widower aged 46).*

Similar to when testing was done during conditions of health concerns or severe illness, it is not very clear, from some women’s accounts, that informed consent was sought and given during routine antenatal testing. Some women even perceived HIV testing during antenatal care to be compulsory, without any opt-out options.

*INTE: How did you come to know that you were HIV positive?*

*VA14: I was never sick; I just went for antenatal care when I was pregnant and was tested at that time.*

*INTE: What did the doctor tell you before?*

*VA14: So when the doctor took my blood is when I knew I had it [HIV].*

*INTE: Did he just take your blood? Or he asked you … telling you he was going to test for what?*

*VA14: You know when we went there we were told it was a must that everybody knows their status, and then he took the blood. (Never married woman aged 22).*

Routine testing of antenatal attendees is meant to identify HIV positive would be mothers so that they receive PMTCT to prevent vertical transmission of HIV. As the narratives show, not all women undertake the test with informed consent. If pregnant women are given the opportunity to opt-out of HIV testing by providers, some are likely to do so. A study in Tanzania showed that about half (49%) of women attending ANC clinics preferred to be given the drugs for preventing vertical transmission rather than learning their HIV status [32].

### HIV testing experience: the ugly

There is evidence from the narratives of testing experience to show that some testing was done without observing any aspect of the 3 Cs principles. Some respondents in this study reported that they were tested without their knowledge and express permission. They also reported that they were neither provided with pre-test counselling, nor post-test counselling:

*INTE: Can you please tell me how you came to know that you were HIV positive?*

*VA08: It was in May 2002. I was pregnant when I got tested at [name of clinic withheld]. But they did not tell me. At that time things were so bad, not as they are right now. These days you are called and counselled well before being told your status. Those who tested me did not tell me anything. They just talked in English thinking that I did not hear what they said, but I did. (widow, aged 36)*

*INTE: Who advised you to undergo HIV testing?*

*KB07: Nobody did. In fact the person who tested me first at [name of clinic withheld] did not even inform me that he was carrying out an HIV test. He just came and told me that I was HIV positive.*

*He asked that I be tested for TB, which was also found to be positive. I was referred to the [name of clinic withheld] where I was tested again and they confirmed the results. (Widower aged 60)*

*INTE: And then what did he (doctor) tell you?*

*VA13: He told me that “now you are like this”. He told me that I had the HIV virus.*

*INTE: Did he counsel you before he told you the results?*

*VA13: He did not counsel me or tell me anything before. He just told me like that.*

Lack of informed consent and counselling prior to or after HIV testing is not only a violation of an individual’s rights, but is also a lost opportunity for behavioural change information to avoid future HIV infection – and if already infected, prevent transmission of the HIV virus to others. The effect of HIV counselling in reducing subsequent risky sexual behaviour has been demonstrated by various studies in SSA [33, 34].

## Discussion

HIV testing in Kenya follows both the VCT and PITC models. This study provides experiential evidence of undergoing an HIV test in both testing regimes in a slum setting in Kenya. There has been simmering concern over how consent, confidentiality and counselling (the 3Cs) can be maintained in resource-limited settings. It provides a sneak peek into the process of HIV testing practice and the challenges faced in maintaining the 3Cs principle.

Both VCT and PITC testing guidelines in Kenya underscore the 3 Cs. The HIV testing and counselling (HTC) guideline state as follows: “prior to receiving an HIV test, the health care provider will explain the procedure and the reasons for requesting the test to the client or patient. Upon the recommendation of the health care provider, if the client or patient agrees to learn their HIV status s/he will receive an HIV test and will be informed of their results” [31]. Counselling is also a core element of international and local testing s in Kenya [31, 35]. These guidelines prescribe both pre- and post- test counselling.

However, as the narratives show, these protocols are not always adhered to in actual practice. Some people undergo HIV testing without their knowledge or express permission. Others report poor quality of care whereby they are either pressurised into testing or they do not receive requisite pre- and post- testing counselling. Our study findings are consistent with related research in this area, albeit on small scale [11, 16, 19].

Not adhering to the 3Cs in either testing model, is not only an infringement of an individual’s rights, but is also a lost opportunity for positive behavioural change communication. Counselling is an opportunity to provide up to date information on HIV prevention and transmission so that people are educated on safe behaviour whether or not they test negative or positive. Evidence suggests that HIV testing and counselling leads to adoption of safer sexual behaviour [27, 36]. Pre-test counselling is meant to provide the individual with sufficient information to help them make an informed choice to undergo an HIV test. Kenyan testing guidelines state that pre-test counselling may be provided to an individual, couple or group, but should be personalised in all cases [35]. A positive test can provoke intense distress, so counselling should help to prepare people for the results and identify sources of medical and social support.

There is always debate between the proponents of the two testing models, with arguments and counter-arguments bringing up tension between human rights and public health imperatives. It is our contention, however, that both the human right and public health imperatives are neither contradictory nor mutually exclusive. As Brockway [37] observes, indeed, “both values can easily be seen as fundamental human good”. It is possible for the HIV testing models to observe both norms, without necessarily undermining either. The narratives show that there is some non-adherence to testing guidelines and principles irrespective of the testing model. What is critical is strict compliance with the three 3Cs irrespective of the testing model. This is especially very critical for the provider-initiated HIV testing of patients seeking medical consultation and antenatal attendees. At the very minimum, the provider must inform the person and make sure that the person understands the procedure he/she will undergo together with attendant implications and consequences. Assuming that consent for testing is implicit by virtue of medical consultation, and hence, can be suspended together with counselling to achieve a public good, is harmful.

### Study limitations

This study is subject to a number of limitations, which must be acknowledged. First, the findings were entirely based on self-reports using face-to-face interviews, which suffers from reporting and social desirability bias. Second, the findings were based on the narratives of the individual respondents who underwent the HIV testing. The views from health providers on the experience of testing and the challenges they faced in maintaining the 3Cs within their daily work environments would have greatly enhanced our study. Third, the study collected information on the HIV positive persons alone, and thus, misses the experiences of those who tested and were HIV negative. Fourth, a few of respondents underwent HIV testing more than a decade ago, and hence, their experiences may not apply now. This is because the field of HIV has seen tremendous improvement in terms of testing algorithms, tailored messaging during counselling, and treatment and care in the last decade. In spite of the aforementioned limitations, the findings offer important lessons about the experiences on HIV testing and the changing landscape in the context of large-scale rapid HIV testing programmes.

## Conclusion

It is concluded that observance of the 3 Cs will ensure that both individual rights are respected and the public good is realised. It is, plausible, to contend that individuals who undergo the HIV test that conforms to the 3Cs principle, will not only feel unharmed (individual good), but will also be more empowered to observe preventive behaviour as well as enrolment to and correct use of ART (public good). Thus HIV testing under conditions of 3Cs is, not only more protective of people’s individual rights, but also for public health good as a more effective HIV intervention tool.

## Data Availability

The datasets analysed during the current study are available from the corresponding author on reasonable request to any scientist wishing to use them for non-commercial purposes, without breaching participant confidentiality and study ethical approval conditions.

## Abbreviations

AIDS: Acquired Immune Deficiency Syndrome
ANC: Antenatal clinic
ART: Antiretroviral treatment
EW: Eliud Wekesa
HIV: Human immunodeficiency virus
IDI: In depth interview
PITC: Provider initiated testing and counselling
PMTCT: Prevention of mother to child transmission of HIV
SSA: sub-Saharan Africa
UNAIDS: Joint United Nations Programme on HIV/AIDS
